# Assessing the use of a mechanical rump pusher in a commercial cattle slaughter plant

**DOI:** 10.1017/awf.2025.10011

**Published:** 2025-06-11

**Authors:** Eleanor Wigham, Megan French

**Affiliations:** School of Biodiversity, One Health and Veterinary Medicine, University of Glasgow, UK

**Keywords:** Animal welfare, automation, bovine, bruising, movement, slaughter

## Abstract

Commercial cattle slaughter operations have shown an increasing trend towards automation, with the aim being to improve animal welfare, product quality and efficiency. Several cattle slaughter plants have introduced mechanical rump pushers (RP) prior to the entrance of the stun box to reduce human-animal interaction and facilitate a smoother transition from the raceway to stun box. Presently, there are no data regarding the use of RPs in commercial slaughter environments operating at 40 cattle per hour. Therefore, this study observed normal operations at a UK slaughter plant, which has an RP installed, and assessed the level of coercion required to enter the RP, the use of the RP, cattle behaviour inside the RP and carcase bruising. The RP was used on 267 of the 815 cattle observed (32.8%) and was more likely to be used on dairy cattle and those who received a higher coercion score when entering the RP. Overall, 60 cattle (7.4%) required the highest coercion score and four (0.49%) required the use of the electric goad. Inside the RP, eleven animals slipped (1.8%) and ten vocalised (1.6%) although no incidences were directly associated with RP use. However, increased time restrained in the RP was significantly associated with more gate slams into the RP entrance gate. The use of the RP was not significantly associated with carcase bruising. These results are encouraging, and although it cannot be concluded that the presence of an RP improves cattle welfare at slaughter, use of automation within cattle slaughter facilities warrants further investigation.

## Introduction

Stress in cattle immediately prior to slaughter is a concern, and can result in poor animal welfare, reduced meat quality (Grandin 2021a) and a decrease in slaughter-line efficiency. Pre-slaughter handling has been described as one of the most stressful events encountered by food-producing animals (Cockram & Corley 1991). Certain human-animal interactions, such as overuse of electric goads, slapping or shouting can negatively impact both animal welfare and meat quality (Warner *et al.* 2007; Waiblinger & Lürzel 2023). Such handling practices are most likely to occur when animals refuse to move forward (Bourguet *et al.* 2011; Hultgren *et al.* 2014). There are numerous reasons why cattle may refuse to move forward, for example, noise, air movement, operators in the animal’s line of sight and inappropriate lighting (Grandin 1996). Animal movement through the slaughter plant can often be improved through raceway design and operator training (Grandin 1993), however, animals may still baulk once improvement measures have been implemented (Grandin 1993).

In particular, the entrance to the stun box can be associated with increased likelihood of cattle baulking and, as a result, increased use of coercion (Grandin 2001; Willson *et al.* 2021). In order to improve cattle movement into the stun box and reduce human-animal interactions, some cattle slaughter plants have installed a mechanical ‘rump pusher’ (RP). These are separate to the stun box and are placed prior to the stun-box entrance. Use of the RP is initiated by an operator when an animal enters the RP but refuses to advance into the stun box. The actuation of the RP involves a metal bar or plate being mechanically lowered behind the rump of the animal and gently pushing it forward into the stun box, thus removing the requirement for other methods of coercion, such as electric goads.

The use of automatic gates to move animals in slaughter plants has been studied in pigs (*Sus scrofa*) (Jongman *et al.* 2021) and results indicate that the minimal handling associated with their use has a positive impact on animal welfare. It has also been reported that reducing human-animal interactions in cattle (*Bos taurus*), especially those unhabituated to handling, can improve welfare (Creamer & Horback 2021). While there has been anecdotal evidence that RPs reduce electric goad use in cattle, currently no published data or literature exist regarding how RPs are used in commercial cattle slaughter operations. Thus the impact of RPs on cattle welfare, meat quality or slaughter-line efficiency remains unclear, as does whether or not coercion is required to enter the RP itself.

This study aims to address this knowledge gap by assessing the use of an RP in a commercial cattle slaughter plant in the UK. Direct observation and review of video footage was used to assess human animal interactions prior to entering the RP and cattle behaviour inside the RP. The presence of carcase bruising was recorded, and the influence of RP use investigated.

## Materials and methods

### Ethical considerations

This study was approved by the University of Glasgow, School of Biodiversity, One Health and Veterinary Medicine Research Ethics Committee (ref EA26/24). It used an observational design and the observers did not interfere with normal operations.

### Study location and slaughter plant design

The study took place at a commercial cattle slaughter plant situated in central Scotland which operates from Monday to Friday. Killing starts at 0700h with the plant processing between 350 to 400 animals daily at an average line speed of 40 animals per hour. Both beef and cull dairy cattle are accepted for processing and a variety of cattle breeds were processed during the study period. The beef cattle consisted primarily of Aberdeen Angus and prime Herefords aged between 18 and 24 months, whilst most dairy cattle were cull Holstein Friesians over 30 months of age.

At the time of the study the slaughter plant was using a stun box (ADL-Thorne Bulltrap Cattle Stun Box, Launceston, UK) which itself contained a hydraulically operated RP (to move the animal forward in the stun box), floor lift, head restraint and side door. Cattle were stunned using a cartridge-fired, captive-bolt gun.

**Figure 1.**
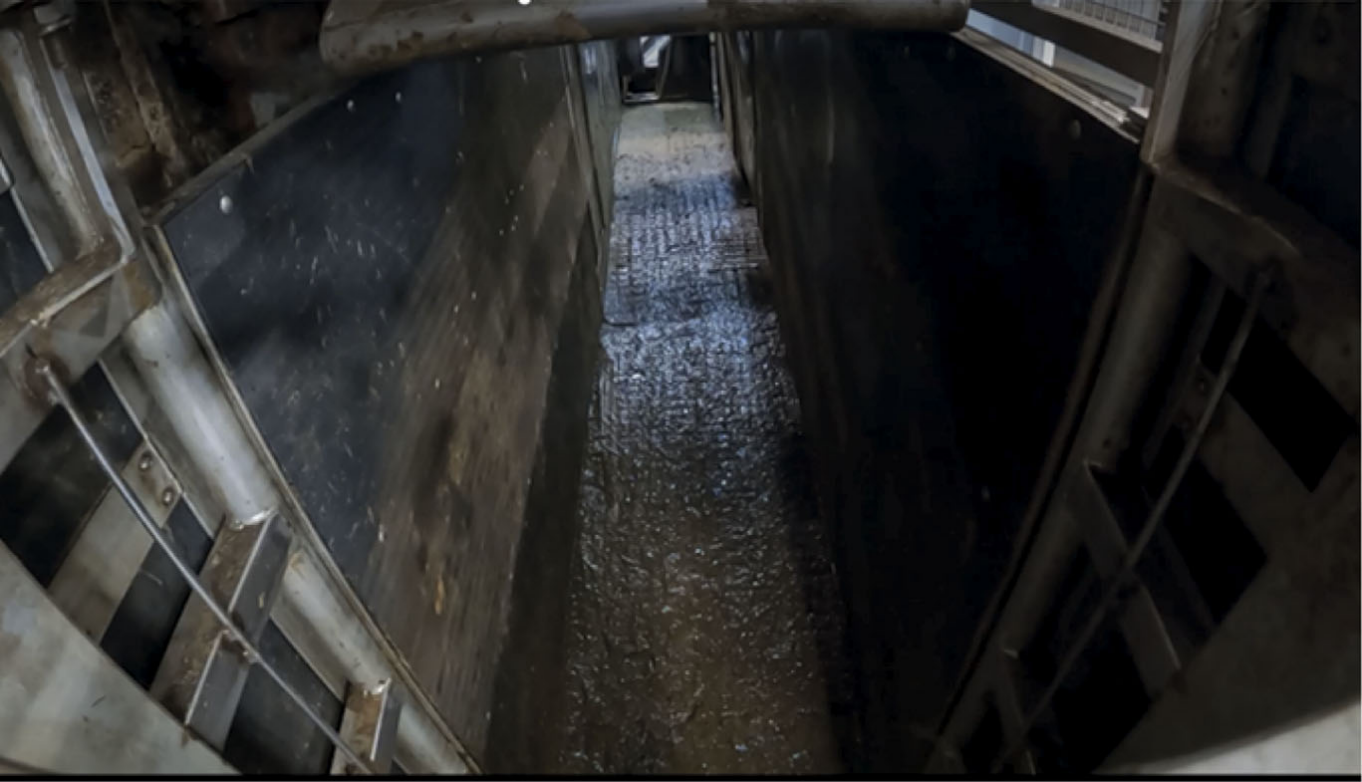
Photographic view from the entrance of the rump pusher (RP) towards the stun box. Both entrance and exit gates of the RP are open with the RP in the upright position.

**Figure 2.**
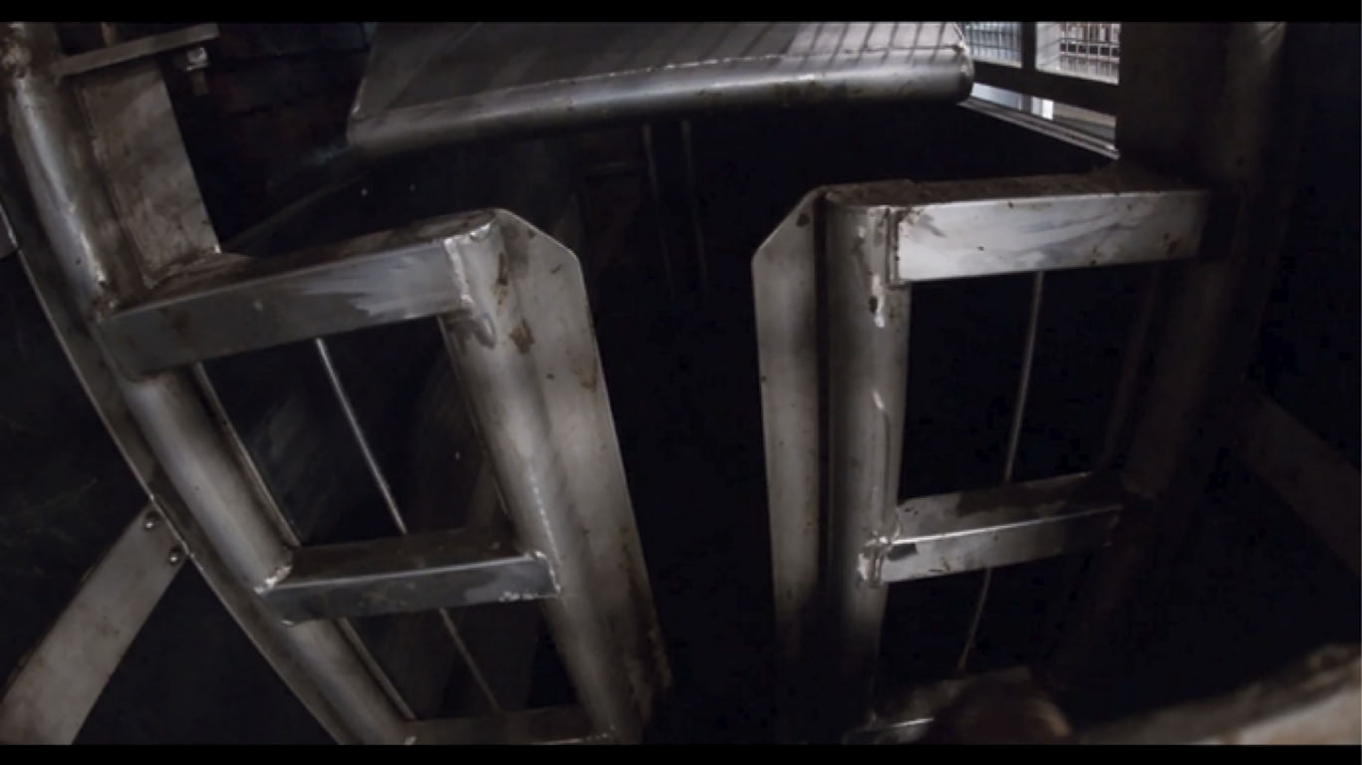
Photographic view from the entrance of the rump pusher (RP) towards the stun box. Both entrance and exit gates of the RP are closed with the RP in the upright position.

The RP (Smisco, Mitchelstown, County Cork, Republic of Ireland) is situated immediately prior to the entrance of the stun box. It is a direct continuation of the raceway, however, is fully enclosed, with a hydraulic gate at both the entrance (from the raceway into the RP) and exit (from the RP into the stun box) (Figures 1 and 2). Only one animal was able to be restrained in the RP at any given time.

**Figure 3.**
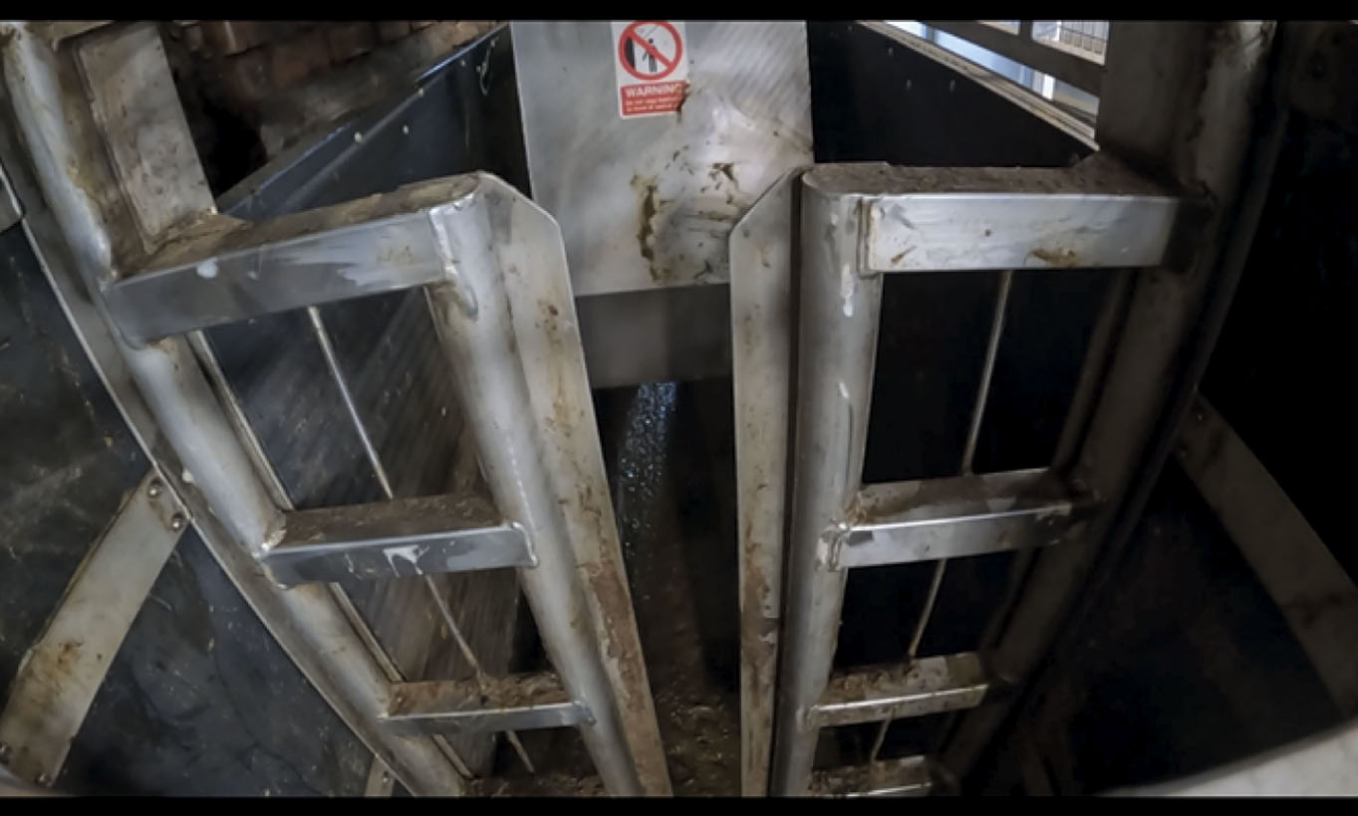
Photographic view from the entrance of the rump pusher (RP) towards the stun box. The entrance gate is closed, and the RP is in the downwards position. From this position the pusher would move forward towards the stun box.

**Figure 4.**
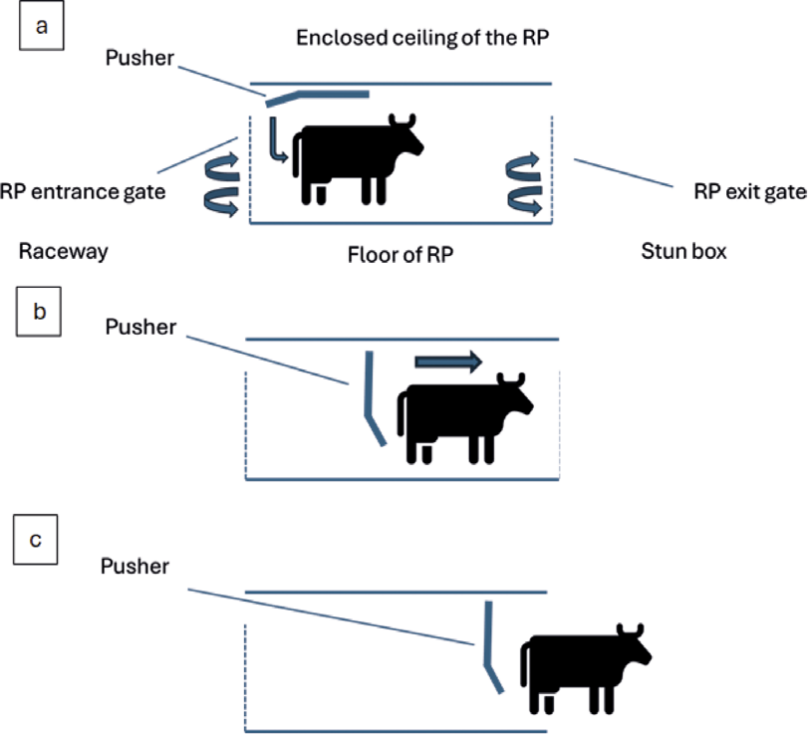
Diagrammatic representation of a side view into the rump pusher (RP). The dashed lines represent the entrance and exit gates both of which are double gates. The entrance gates open outwards into the raceway (represented by arrows in [a]; see Figures 1 and 2). The exit gates open inwards into the RP (represented by arrows in [a]). Once the RP is initiated it lowers down behind the animal (a) and pushes it forward ([b] and Figure 3) until it has left the RP and entered the stun box (c). After which point the RP exit gates close, and the pusher returns to the upright position (a). The forward movement of the pusher can be stopped at any point by the operator.

Use of the RP is initiated and controlled by an operator when an animal enters the RP box but refuses to advance into the stun box. In such instances the metal ‘pusher’ is hydraulically lowered behind the rump of the animal and then moves it forward (Figures 3 and 4). The forward movement of the pusher can be stopped at any point by the operator. During observations a distinction was made between the full use of the RP, when the pusher remains in contact with the animal until it has entered the stun box, and an RP ‘touch’ when the animal moved forward immediately on contact, and use of the RP terminated prior to further contact with the animal (Table 1). The exact measurements of the stun box and RP are unknown since direct access to these areas was not possible.

**Table 1.** Welfare assessment measures and associated scoring system used by observer A positioned at the rump pusher (RP)

Measure	Definition	Scoring
Coercion	Interaction between slaughter plant operative and animal whilst moving into the RP.	No human animal interaction. Use of hand motion (no contact with animal) and or voice at normal volume/intensity. Gentle touch with hand onto animal (causing no audible sound). Slapping animal (causing audible sound), shouting, use of a tool. Adapted from (Jones 2011)
Electric goad	The electric goad was used on the animal to enter the RP.	Yes No
Type of animal	Breed of animal.	B. – Beef Breed D. – Dairy Breed
Use of RP*	The RP was initiated and contacted animal.	No – RP was not used. Touch – RP was used, animal moved forward immediately on contact, and use of the RP terminated prior to further contact with the animal. Yes – RP was used to push animal into the stun box. The pusher remains in contact with the animal until it has entered the stun box
Slips*	Slips and loses balance temporarily, interfering with normal walking (Maria *et al.* 2004). Scored inside RP.	Yes (number of slips/whether slip occurred when RP was in use) No
Vocalisation	Animal vocalised inside the RP.	Yes (whether vocalisation occurred when RP was in use) No
Gate slam*	Whilst inside the RP the animal’s rump contacts the closed RP entrance gate (see Figure 2) with enough force to make an audible sound. Animal must take two steps forward before a subsequent contact is scored.	Yes (number of times contacted) No
Time inside the RP*	Time from the closure of the RP entrance gate until the animals moves inside the stun box and the RP exit gate is closed.	Seconds

* Measure observed retrospectively using the GoPro footage.

### Observations

Data collection took place daily over a two-week period in the summer of 2024. Observations of normal slaughter plant practice primarily occurred between 1000 and 1200h each day, however there was some variation to account for recharging of camera batteries and line breakdowns. During the data collection period every animal entering the RP was observed and scored.

**Figure 5.**
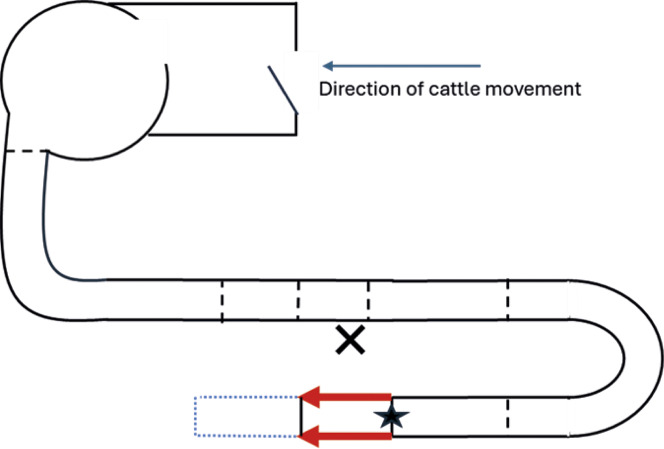
Diagrammatic representation of the raceway in which cattle are moved from the lairage pens to the slaughter point. Dashed lines represent gates, X represents location of Observer A, the parallel arrows represent position of the rump pusher (RP), and the dotted rectangle represent the stun box. The GoPro camera was located at the ★ angled towards the stun box.

A trained observer (A) was positioned in the lairage at close proximity to the RP (Figure 5). As the RP had solid sides, precluding direct observation, a GoPro camera (Hero10, GoPro Ltd, San Mateo, CA, USA) was attached above the entrance gate to the RP to enable filming of the cattle inside the RP.

The observations collected by observer A and from review of the GoPro footage are shown in Table 1.

A second trained observer (B) was positioned inside the slaughter hall after the dehiding process and prior to carcase splitting. This enabled clear observation of the entire carcase for bruise scoring. The same individuals acted as observer A and observer B throughout the entire data collection period.

**Figure 6.**
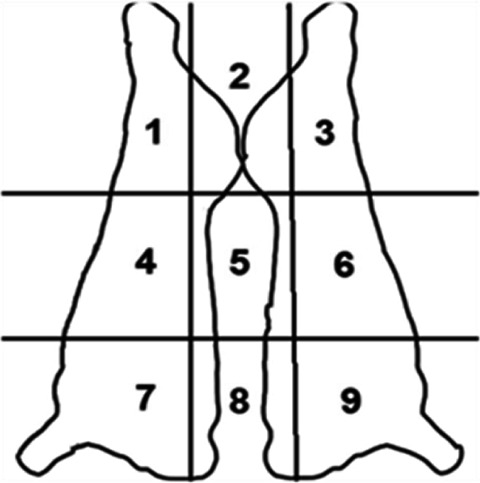
Carcase outline with bruise scoring grid used by observer B. Only bright red bruises in sections 1, 2 or 3 were recorded.

Carcases were assessed for the presence of rump bruising and scored using a modified version of the scoring system described by Lee *et al.* (2017). Carcases were scored as ‘bruised’ if they had one or more visible bruises in the rump and hind leg area of the carcase (areas 1, 2 and 3 shown in Figure 6). Size and severity of the bruise was not recorded as direct access to the carcase to facilitate measurement was not feasible. Gracey and Collins (1992) described that the age of a bruise can be estimated from its colour appearance in bovine carcases; a bright red bruise is likely to be up to 10 h old, whereas a dark red bruise is approximately 24 h old. Here, in an effort to record bruising which may have occurred at the slaughter plant, only bright red bruises were scored.

Correlation between animals observed in the RP and in the slaughter hall was maintained by using the kill number allocated by the slaughter plant.

### Statistical analysis

Observational data from the RP and carcase bruising were edited and analysed using Excel® (version 2410; Microsoft Corp, Redmond, WA, USA) and SPSS® (version 26; IBM Corp, Armonk, NY, USA).

For statistical analyses the use of the RP was scored as either ‘no’ or ‘yes’ with ‘yes’ including observations of ‘yes’ and ‘touch’.

A binomial logistic regression model was used to analyse whether coercion score and type of animal (independent variables) could predict whether the RP would be used (dependent variable). To create a binomial dependent variable, an observation of ‘touch’ during RP use was classified as use of the RP. A binomial logistic regression model was also used to assess the effect of RP use, coercion score, cattle type and gate crashes (independent variables) on the risk of carcase bruising (dependent variable). The Nagelkerke R-square, the Hosmer and Lemeshow test were used as measures of the model’s goodness of fit. The Wald test was used to determine statistical significance (*P* < 0.001) for each of the independent variables in each of the models. Beef cattle and coercion score 0 were arbitrarily chosen as base levels for all regression models.

A Mann-Whitney *U* test was used to test for associations between time an animal spent in the RP and use of the RP. A negative binomial regression model was used to assess the effect of the time spent in the RP (independent variable) on the risk of an animal contacting the gate (dependent variable). This type of regression is used to model count data when there is evidence of overdispersion, and the data do not follow a Poisson distribution.

## Results

A total of 815 cattle were directly observed moving through the RP into the stun box, 664 (81.5%) were beef type and 151 (18.5%) were dairy type.

### Entering and use of the RP

The RP was used on 267 (32.8%) of cattle, with dairy cattle (91; 60.3%) seeing more usage than beef (76; 26.5%) (Table 2).

**Table 2.** Number of times the rump pusher (RP) (n = 815) was used by cattle type

RP use	Total (% of all cattle)	Beef (% of beef cattle)	Dairy (% of dairy cattle)
Yes	183 (22.5%)	119 (17.9%)	64 (42.4%)
Yes (Touch)	84 (10.3%)	57 (8.6%)	27 (17.9%)
No	548 (67.2%)	488 (73.5%)	60 (39.7%)

When entering the RP, the majority of animals (468; 57.4%) required no or very gentle coercion (score 0 or 1) whilst 60 animals (7.4%) required the highest coercion score of 3. Of the animals requiring a coercion score of 3 to enter the RP over half (34; 56.7%) required use of the RP to enter the stun box (Table 3). The electric goad was used on four animals (0.49%) whilst entering the RP, all of which were beef breeds. The RP was used on all these animals.

**Table 3.** Number of times each coercion score (see Table 1) was used when cattle (n = 815) were entering rump pusher (RP) by cattle type and subsequent RP use

Coercion score	Total (% all cattle)	Beef (% of beef cattle)	Dairy (% of dairy cattle)	RP used (% within coercion score)
0	135 (16.6%)	116 (17.5%)	19 (12.6%)	28 (20.7%)
1	333 (40.9%)	278 (41.9%)	55 (36.4%)	82 (24%)
2	287 (35.2%)	219 (33%)	68 (45%)	123 (42.9%)
3	60 (7.4%)	51 (7.7%)	9 (6%)	34 (56.7%)

The results of the binomial regression model exploring the effect of coercion score and animal type on RP use are shown in Table 4. The model was statistically significant χ^2^(4) = 104.184; *P* < 0.001, explained 16.7% (Nagelkerke R^2^) of the variance in RP use and correctly classified 70.8% of observations. Compared to beef cattle, dairy were 4.24 (95% CI: 2.89–6.20) times more likely to require RP use. Animals requiring coercion scores 2 and 3 to enter the RP were 2.66 (95% CI: 1.62–4.36) and 5.46 (95% CI: 2.77–10.77) times more likely, respectively, to require RP use.

**Table 4.** Results of the binomial logistic regression for effect of coercion score and animal type on rump pusher (RP) use

Variable	OR	CI-95%	Wald test *P*-value
**Coercion score**			
0	*Ref*		
1	1.21	0.73–2.00	0.451
2	2.66	1.62–4.36	**< 0.001**
3	5.46	2.77–10.77	**< 0.001**
**Animal type**			
Beef	*Ref*		
Dairy	4.24	2.89–6.20	**< 0.001**

OR: Adjusted Odds ratio, CI: Confidence interval. Bold denotes significance.

### Observations inside the RP

Of the total of 609 cattle filmed inside the RP, 510 (83.7%) were beef and 99 (16.3%) were dairy types. The RP was used on 202 (33.2%) of these animals. Discrepancies between the number of animals filmed and directly observed were due to battery capacity and the inability to change the GoPro camera during production.

Eleven (1.8%) animals slipped at least once inside the RP and all these animals were beef types with the RP used on one individual. The highest number of slips observed per individual animal was three with no slips observed whilst the RP was in use.

Ten (1.6%) animals (two dairy, eight beef) vocalised inside the RP. Of these, two required RP use (one dairy, one beef). No vocalisations were recorded whilst the RP was in use.

For all animals observed (n = 609), the mean (± SD) time spent inside the RP was 61.56 (± 42.52) s, the median 61 s, and the range 2–639 s (the reason for such a prolonged time-period within the RP was due to an animal being the first to be slaughtered after a routine break in processing. The animal in question was moved into and contained within the RP by the lairage operators prior to the return of the operator responsible for shooting the cattle).

The difference in time spent in the RP between animals that required RP use (n = 202; median time: 66 s) and those who did not require RP use (n = 407; median time 58 s) was statistically significant (z = –4.47; *P* < 0.001).

The majority (405; 66.5%) of cattle performed at least one gate slam whilst inside the RP. Ninety-seven cattle (15.9%) performed five or more gate slams whilst 12 (2.0%) performed ten or more. The highest number of gate slams performed by an individual animal was 14. Time spent in the RP box positively predicted the number of gate slams (IRR = 1.016, 95% CI: 1.013–1.019; *P* < 0.001) (Model χ^2^ [1] = 6.074; *P* = 0.014).

### Bruising

A total of 668 carcases were scored for bruising, 536 (80.2%) were beef and 132 (19.8%) dairy. The RP was used on 223 (33.4%) of the carcases scored. Bruising was detected on 286 (42.8%) of carcases, 96 (72.7%) of dairy cattle had bruising compared to 190 (35.5%) of beef cattle. Of the cattle which required RP use (n = 223), 119 (53.4%) had bruising present (Table 5).

**Table 5.** Results of carcase bruise scoring by animal characteristic

	Total	Dairy Type	Beef Type	Rump pusher used	Rump pusher not used	Coercion score 0	Coercion score 1	Coercion score 2	Coercion score 3
Number of carcases	668	132	536	223	445	107	273	237	51
Number of carcases with visible bruising	286	96	190	119	167	49	102	108	27
% of carcases with visible bruising	42.8%	72.7%	35.5%	53.4%	37.5%	43.0%	37.4%	45.6%	52.9%

A complete data set of RP use, coercion score, animal type, gate slams and carcase bruising were available for 560 animals. The results of the binomial regression model exploring the influence of RP use, coercion score, animal type, gate slams on carcase bruising are shown in Table 6. The model explained 10.9% (Nagelkerke R^2^) of the variance in RP use and correctly classified 65.7% of observations, however, was not statistically significant χ^2^ (8) = 4.47; *P* = 0.812. Compared to beef cattle, dairy were 3.67 (95% CI: 2.22–6.08) times more likely to have carcase bruising. Animals that required a coercion score of 1 to enter the RP were significantly less likely to have carcase bruising than those requiring a coercion score of 0. RP use, gate slams and coercion scores of 2 and 3 did not have a significant impact on the likelihood of carcase bruising (Table 6).

**Table 6.** Results of the binomial logistic regression exploring the influence of rump pusher (RP) use, coercion score, animal type and gate slams on carcase bruising

Variable	OR	CI-95%	Wald test *P*-value
**Coercion score**			
0	*Ref*		
1	0.51	0.30–0.85	**0.01**
2	0.68	0.40–1.15	0.15
3	1.23	0.57–2.65	0.61
**Animal type**			
Beef	*Ref*		
Dairy	3.67	2.22–6.08	**< 0.001**
**RP use**			
No	*Ref*		
Yes	1.42	0.95–2.12	0.09
**Gate Slam**	1.043	0.97–1.12	0.25

OR: Adjusted Odds ratio, CI: Confidence interval. Bold denotes significance.

## Discussion

Transfer of an animal from the lairage pen to the stunning area is a key point for animal welfare consideration in a slaughter plant (EFSA 2020). The direct handling of animals by humans, when inappropriate, can be harmful to welfare and meat quality (Pajor *et al.* 2000; Warner *et al.* 2007). Installation of an RP prior to the stun box can be a solution in maintaining cattle throughput rate, whilst reducing human-animal interactions. However, there are a paucity of studies assessing the impact of RPs on welfare in commercial cattle slaughter plants. This study sought to address this gap by evaluating the use of an RP in such a plant using predominantly animal-based measures.

Overall, the RP was used on one-third (267; 32.8%) of cattle in the study population, however a proportion of its use (84; 31.5%) was classified as a ‘touch’ where the use of the RP was terminated prior to completion of a full push. Often this was due to the animal having moved forward into the stun box and therefore not requiring further coercion. The RP is manually controlled by an operator with the decision to either activate the RP or terminate prior to a full push likely multifactorial and potentially taking into account both animal- and situational-based considerations.

### Coercion

It is unsurprising that a higher proportion of dairy cattle (91; 60.3%) required the RP compared to beef cattle (176; 26.5%). Dairy cattle are habituated to human handling, which can result in increased tameness and reduced fearfulness towards people (Probst *et al.* 2012), leading to no flight zones (Ewbank & Parker 2024). However, when entering the RP similar proportions of beef (51; 7.7%) and dairy (9; 6%) cattle required the highest coercion score (slapping animal causing audible sound, shouting, use of a tool). Higher coercion scores of 2 or 3 were associated with increased odds of RP use, likely reflective of these animals’ general reluctance to move forward. Although flags and paddles are considered good alternatives to prevent the use of electric goads (EFSA 2020), understanding the reasons why cattle are reluctant to move (often due to poor design or distractions; Grandin 2024) and implementing corrective measures, are key aspects of welfare improvement measures in all slaughter plants.

In this study, the electric goad was used on four animals (0.49%) each time during entry to the RP. The purpose of an electric goad is to cause discomfort or pain and, consequentially, a movement response in an animal (Whiting 2016; Grumett & Butterworth 2022). Welfare concerns have led retailers in the UK to apply pressure to the slaughter industry to reduce use of electric goads on livestock (Wigham *et al.* 2018). Compared to other published work, the use of the electric goad in the study plant was relatively infrequent, for example, work conducted in Ecuador reported that all cattle at the study slaughter plant were prodded with an electric goad (Cevallos-Almeida *et al.* 2021) and a Mexican slaughter facility reported that 67% of the over 8,000 cattle studied were prodded with the electric goad during the stunning stage (Miranda-de la Lama *et al.* 2012). In Europe, reports from a commercial Swedish slaughter plant show that the goad was used on 20.1% of cattle (Hultgren *et al.* 2020), and a French study reported that cattle received, on average, 7.1 (± 0.2) electrical prods during their time at the study slaughter plant (Bourguet *et al.* 2011). Although it is not possible to conclude that the RP is the primary cause of the relatively low incidence of electric goad use, it is encouraging that installation of an RP is not associated with excessive coercion methods.

### Behaviours inside RP

It was observed that cattle were often moved into and restrained in the RP whilst the preceding animal was still in the stun box, and therefore the RP exit gates were closed. This practice reduced the opportunity for animals to move directly through the RP and likely contributed to a median time spent in the RP of just over a minute (61 s). The positioning of the GoPro camera unfortunately obstructed the view of the RP exit gate during containment of an animal within the RP. It was not possible, therefore, to collect data regarding any delay between the RP exit gates opening and any RP activation. Consequently, it is unknown for how long cattle would baulk once the RP exit gates were open and before the RP was activated. A significant correlation was found between increased time spent in the RP and increased odds of gate crashes. Restraint is a cause of significant stress in cattle (Chen *et al.* 2016), moving backwards can be used as an animal-based measure for ‘fear’ (EFSA 2020) and bumping into solid structures has been reported as a significant risk factor in carcase bruising (Hoffman & Lühl 2012) (although a significant relationship was not found in this study). Operators should therefore consider the length of time that an individual animal is restrained in the RP and continue to evaluate this with respect to maintaining the required levels of throughput.

Slipping, whereby an animal temporarily loses its footing, can cause cattle to become agitated (Grandin 1998). Slipping is indicative of slippery flooring on which animals risk more serious falls and injury (EFSA 2020) and therefore should be avoided. Eleven animals (1.8%) were observed slipping whilst inside the RP, however none of these slips were deemed to be a direct cause of the use of the RP. Slipping incidence was not recorded in any other areas of the slaughter plant, therefore it is unknown whether being contained inside the RP resulted in more slipping compared to being contained in the raceway or stun box. The incidence in this study lies within The North American Meat Institute (NAMI) audit criterion of less than 3% of animals observed slipping (Grandin 2021b). Similarly, the percentage of cattle recorded to have vocalised in this study (1.6%) is also within the NAMI audit criterion of 5% or less (Grandin 2021b). There is evidence that cattle vocalisation scoring can be used to identify areas of severe welfare compromise in a slaughter plant (Grandin 1998, 2001; Bourguet *et al.* 2011), however, it should be noted that cattle are capable of vocalising in a variety of states other than that of adverse welfare (MacKay *et al.* 2014). No vocalisations were recorded whilst the RP was in use, and the vocalisations were not associated with any other behaviours, such as slipping or gate slams, therefore the reason for the vocalisations is unknown, and could be explored further.

### Bruising

Although this study did not find any significant correlation between RP use and bruise scoring (OR = 1.42, 95% CI 0.95–2.12; *P* = 0.09), the RP was used on 119 (41.6%) of the 286 carcases showing bruising. Due to the animal welfare and economic consequences of carcase bruising (Wigham *et al.* 2018), further work exploring any potential association with RP use is warranted.

A bruise is caused by vascular rupture, leading to blood accumulation in the muscle and other tissues as a result of impact from an animal’s environment, a conspecific or due to human-animal interactions (Costa *et al.* 2006). Strappini *et al.* (2013) concluded that human-animal interactions at the slaughter plant, in particular during unloading and at stunning, created the greatest potential for traumatic events. The rough handling of animals, and the use of driving instruments (prods, sticks, whips) pre-slaughter, shows a positive correlation with increased levels of bruising (Jarvis *et al.* 1995; Huertas *et al.* 2010). However, there are a number of factors that can affect carcase bruising prevalence including: loading and unloading (Strappini *et al.* 2013); transport conditions; horned animals being present (Huertas *et al.* 2010); movement through markets; animal sex, age (Weeks *et al.* 2002; Özdemir & Ekiz 2023) and breed (Lee *et al.* 2017). Although colour can be used to estimate the age of a bruise (Gracey & Collins 1992), it is not possible to determine exactly when the damage occurred, and it should be noted that bruising can occur post-stun, for example, during roll out from the stun box (blood pressure is maintained prior to the thoracic stick). Previous studies at UK slaughter plants have reported bruising prevalence ranging from 59% (McNally & Warriss 1996) to 97% of carcases (Jarvis *et al.* 1995). Prevalence in this study was 42.8% however only bright, haemorrhagic red bruises were recorded (since these are likely to be 0–10 h old; Gracey & Collins 1992), and only those present in the rump and hind leg area of the carcase (as it was reasoned that any bruising caused by the RP would most likely be fresh and present on the hindquarters). The binomial logistic regression (Table 6) was not significant, which may reflect the numerous factors known to influence carcase bruising that were not included in the data collection, and therefore not included in the statistical model. Dairy cattle were significantly more likely to have bruising than beef cattle, which aligns with the results of a recent meta-analysis (Sánchez *et al.* 2022) and could be linked to sex (Bethancourt-Garcia *et al.* 2019), age (Šímová *et al.* 2016), body condition score (BCS) (Sánchez-Hidalgo *et al.* 2019) and hormonal influence (Riley *et al.* 2014). It is not known why cattle with a coercion score of 0 had a greater likelihood of carcase bruising than those with a score of 1. Additional information regarding individual cattle characteristics may have helped explain this result, however it was not possible to collect information regarding age/sex/BCS etc in this study.

Since direct access to the carcase was not feasible, exact size measurement of bruising was not possible. For ease of scoring, carcases were classified as either bruised or not bruised, with the extent of the bruising not considered. Visual assessment of bruising has been found to often underestimate the amount of trimming required during carcase dressing with bruises tending to have an ‘iceberg effect’ whereby visual carcase bruising seen on the surface does not accurately represent the magnitude of bruising within the tissue (Kline *et al.* 2020). The scoring system used, and the iceberg effect may therefore have underestimated the bruising prevalence. A more sophisticated system to age and score bruises could be beneficial in determining their origin and potential welfare and economic impact in any future studies.

### Study limitations

There are a number of methodological limitations to this study that must be considered. Only one slaughter plant was included which was selected, to a great extent, as a result of its availability and willingness to participate. It was not possible to assess welfare, or access welfare records from prior to RP installation. Therefore, the results cannot conclude causality as to whether having an RP installed improves welfare or efficiency at a slaughter plant. There is the potential that the physical presence of an observer during welfare assessments can affect the behaviour of processing plant personnel who ‘improve their performance’ during the observation period, but revert back to normal practice when they are no longer being watched (Grandin 2010). This ‘Hawthorne effect’ (the alteration of behaviour by the subjects of a study due to their awareness of being observed) may have positively influenced the results of the welfare assessment. Reduced battery life of the GoPro camera impacted the collection of data. The camera could not be replaced part-way through data collection, this was due to health and safety risks and to prevent disruption of normal production.

## Animal welfare implications and Conclusion

The use of technology and the extent of automation throughout the slaughter process is increasing (Kim *et al.* 2023). There is evidence from pig studies that the use of automatic gates is associated with welfare advantages due to the minimal stress caused by handling (Jongman *et al.* 2021; Velarde & Dalmau 2024). This was the first attempt to collect information on the use of automation to move cattle in lairages. Although it cannot be concluded from this study alone that the presence of an RP improves cattle welfare at slaughter, its findings are encouraging. The animal-based measures of welfare assessed in this study are within the limits outlined by industry guidance and in some cases, such as the need for higher levels of coercion, indicate improved levels of welfare compared to other published work. Furthermore, no animal-based measure of welfare, namely slipping, vocalisation or bruising, was directly associated with the RP being used. More data are needed to better understand how automation could be used to enhance welfare, product quality and efficiency in cattle at the time of slaughter.
